# Duodenum-Preserving Resection of the Pancreatic Head versus Pancreaticoduodenectomy for Treatment of Chronic Pancreatitis with Enlargement of the Pancreatic Head: Systematic Review and Meta-Analysis

**DOI:** 10.1155/2017/3565438

**Published:** 2017-08-22

**Authors:** Yajie Zhao, Jianwei Zhang, Zhongmin Lan, Qinglong Jiang, Shuisheng Zhang, Yunmian Chu, Chengfeng Wang

**Affiliations:** Department of Abdominal Surgical Oncology, National Cancer Center, Cancer Hospital, Chinese Academy of Medical Sciences and Peking Union Medical College, Beijing 100021, China

## Abstract

The results of this meta-analysis show that DPPHR should be established as first-line treatment because of lower level of severe early postoperative complications, maintenance of endocrine pancreatic functions, shortening of postoperative hospitalization time, and increase of quality of life compared to pancreaticoduodenectomy.

## 1. Introduction

Chronic pancreatitis (CP) is defined as “A continuous inflammatory process causing permanent structural damage to the pancreatic gland, which ultimately results in impairment of the gland's exocrine and endocrine function” [1]. About 30% of CP patients have inflammatory enlargement of the pancreatic head (PH) [2]. PH enlargement can result in stenosis of the common bile duct or obstruction of the main pancreatic duct [3]. Hence, patients with inflammatory enlargement of the PH usually require PH resection to resolve these complications. The traditional Whipple procedure is first-line therapy for this type of CP, but it is associated with a high prevalence of morbidity and mortality and reduced quality of life (QoL) [4, 5]. These disadvantages are attributed to extensive resection, including the removal of the duodenum and a large portion of the pancreas. The duodenum plays an important part in the regulation of digestive processes. In 1990, Beger and Buchler introduced duodenum-preserving pancreatic head resection (DPPHR) to preserve the duodenum and limit resection of pancreatic tissue [6]. A similar procedure was proposed by Ho et al. in which local resection of the PH with longitudinal pancreaticojejunostomy is carried out [7]. The literature suggests that both of these procedures are suitable for the treatment of this type of CP. Several studies have discussed the potential superiority of DPPHR over other surgical methods, but a systematic and quantitative review summarizing the available evidence is lacking. Therefore, we undertook a meta-analysis to compare the effectiveness and safety of DPPHR versus pancreaticoduodenectomy (PD) or pylorus-preserving pancreaticoduodenectomy (PPPD) for CP.

## 2. Methods

**Figure 1:**
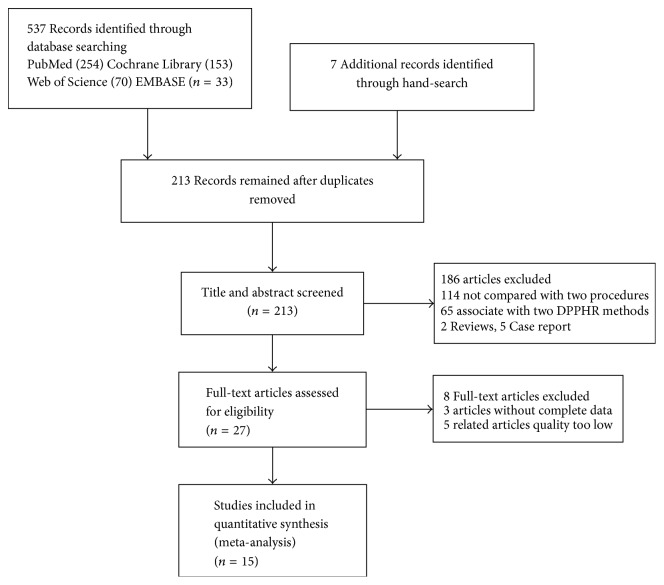


We searched for journal articles published from January 1990 to September 2016 by electronic and manual means. We searched the databases of PubMed, the Cochrane Library, Web of Science, and EMBASE using the following search terms: (pancreaticoduodenectomy OR duodenopancreatectomy OR pancreatectomy OR pylorus-preserving OR Whipple or PD or PPPD) AND (duodenum-preserving pancreatic head resection OR duodenum-preserving OR Beger or longitudinal pancreaticojejunostomy OR longitudinal pancreaticojejunoanastomosis OR LPJ OR Frey OR Beger OR DPPHR) AND (chronic pancreatitis). A language restriction was not applied and the search was carried out by two independent investigators (Figure 1).

### 2.1. Inclusion Criteria

We searched for studies comparing DPPHR with PD or PPPD for CP with inflammatory enlargement of the PH. We also searched for studies in which elective surgery was planned for patients diagnosed with CP in the PH. If the same study had been published more than once then the latest publication was used.

### 2.2. Exclusion Criteria

A study was excluded if (i) the method of surgery was not reported; (ii) a comparison between the DPPHR group with a PD group or PPPD group was not made; (iii) the prevalence of postoperative complications and mortality as study outcomes was not reported; (iv) the study had been reported before; (v) it had design flaws and was of low quality; (vi) it was an abstract, case report, letter, comment, or review without original data; (vii) if it presented insufficient data.

### 2.3. Literature Screening

All literature was screened by two independent investigators. If the two investigators disagreed, then they tried to resolve the disagreement through discussion. If discussion failed, the final decision was made by a third investigator. EndNote reference management software was used to search and remove duplicate studies.

### 2.4. Data Extraction

The following detailed data were extracted independently by the two investigators and checked by the other authors: title; authors; year of publication; country; study design; surgery type; number of patients (age, sex); postoperative factors (delayed gastric emptying, endocrine/exocrine insufficiency, duration of hospitalization, pain relief, pancreatic fistulae [Grade B + C], wound infection, and mortality).

### 2.5. Statistical Analyses

Review Manager v5.3.0 (Cochrane Collaboration) was used to carry out the meta-analysis in accordance with the PRISMA statement. Odds ratios (ORs) were used for the analyses of dichotomous variables and 95% confidence intervals (CIs) values are reported. The Mantel–Haenszel, chi-square, and *I*^2^ tests were used to ascertain the heterogeneity between studies. *I*^2^ < 50% suggested that the heterogeneity was not significant, and consequently a fixed effects model was used. *I*^2^ > 50% suggested significant heterogeneity, and consequently a random-effects model was applied. *P* < 0.05 was considered significant. Funnel plots were used to assess a potential publication bias.

### 2.6. Characteristics of Included Studies and Quality Assessment

On the basis of the inclusion and exclusion criteria, 15 studies (eight randomized clinical trials (RCTs) and seven retrospective cohort studies) were included in this meta-analysis. These 15 studies involved 1586 patients (797 in the DPPHR group and 789 in the DP/PPPD group). The detailed characteristics of all included studies are shown in Table 1.

The quality of RCTs was evaluated based on the Jadad scale, which was used to assess randomization, concealment of allocation, blinding, and withdrawals in each study. Each item was given a score of 0–2, and the maximum total score was 7. If the total score was ≥4, the RCT was of “high” quality. Observational clinical studies (OCS) were scored based on the Newcastle–Ottawa system, which involves assessment of selection, comparability, and exposure/outcome. The maximum total score was 9. If the total score was ≥7, the OCS was considered to be of “high” quality.

### 2.7. Assessment of the Risk of Bias of RCTs

**Figure 2:**
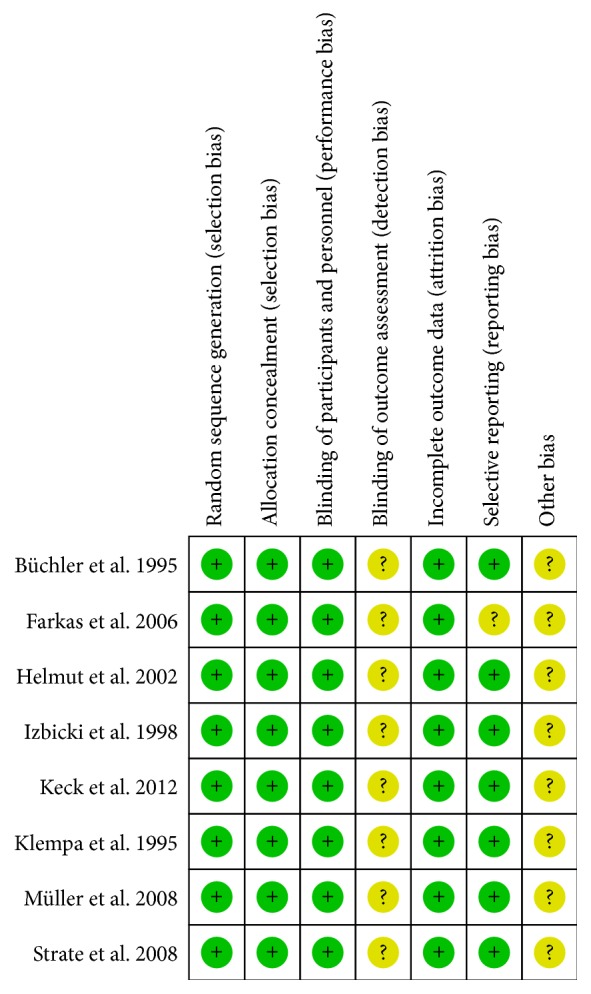


For the included RCTs, assessment of the bias risk involved six parameters: allocation concealment, incomplete outcome data, blinding, selective reporting bias, sequence generation, and other potential sources of bias. Assessment was based on a quality checklist recommended in the Cochrane Handbook. “Yes” indicated a “low” risk of bias; “unclear,” an “uncertain” risk of bias; “no,” a “high” risk of bias (Figure 2).

### 2.8. Meta-Analysis Results

#### 2.8.1. The Rate of Pain Relief

**Figure 3:**
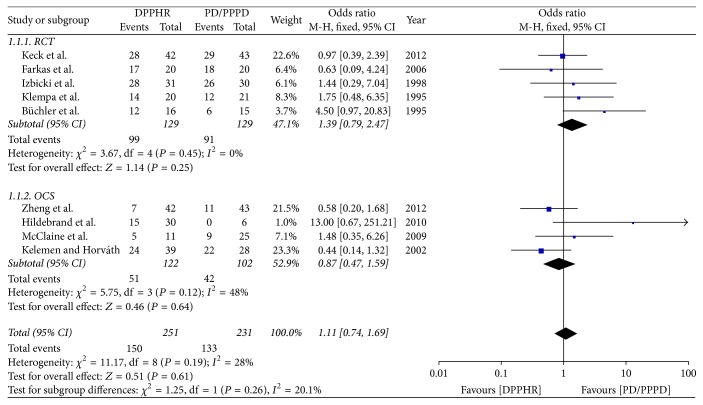
Meta-analysis of the rate of pain relief.

Nine included studies reported the rate of pain relief; we pooled data from the nine studies to compare DPPHR group with PD/PPPD group. The results of meta-analysis show that there is no difference between two groups in the rate of pain relief (OR = 1.11; 95% CI, 0.74–1.69; *P* = 0.61; *I*^2^ = 28% for heterogeneity), Therefore, using a fixed model, the meta-analysis of RCTs and OCS subgroup reveals the rate of pain relief was not statically different between two groups [RCTs (*I*^2^ = 0%, OR = 1.39; 95% CI, 0.79–2.47; *P* = 0.25), OCS (*I*^2^ = 48%, OR = 0.87; 95% CI, 0.47–1.59; *P* = 0.64)] (Figure 3).

#### 2.8.2. Incidence of Pancreatic Fistula

**Figure 4:**
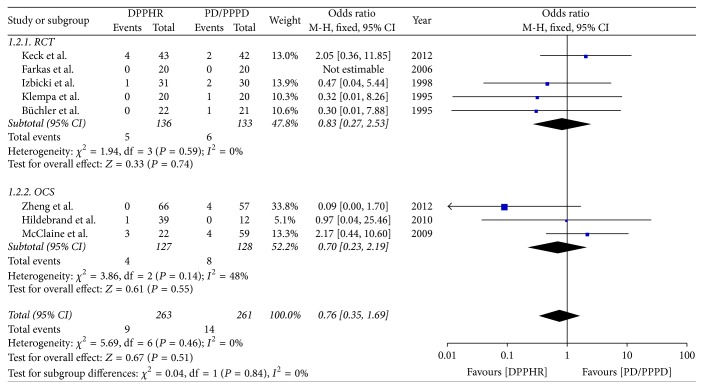
Meta-analysis of the incidence of pancreatic fistula.

Eight included studies reported the incidence of postoperative pancreatic fistula; we pooled data from the eight studies to compare DPPHR group with PD/PPPD group. The results of meta-analysis show that there is no difference between two groups in the rate of POPF (OR = 0.76; 95% CI, 0.35–1.69; *P* = 0.51; *I*^2^ = 0% for heterogeneity). Therefore, using a fixed model. The meta-analysis of RCTs and OCS subgroup reveals no heterogeneity among studies and the incidence of POPF was not statically different between two groups. [RCTs (*I*^2^ = 0%, OR = 0.83; 95% CI, 0.27–2.53; *P* = 0.74), OCS (*I*^2^ = 48%, OR = 0.70; 95% CI, 0.23–2.19; *P* = 0.55)] (Figure 4).

#### 2.8.3. Incidence of Wound Infection

**Figure 5:**
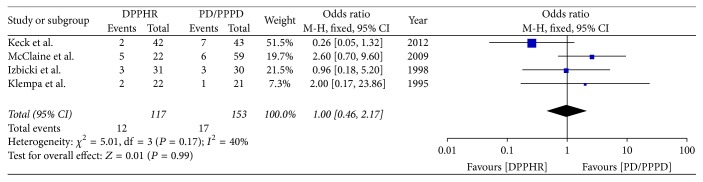
Meta-analysis of the incidence of wound infection.

Four included studies reported the incidence of wound infection after PD/PPPD and DPPHR. *I*^2^ (*I*^2^ = 40%) revealed no obvious heterogeneity among these studies; therefore using a fixed model, there was no significant difference between DPPHR and PD/PPPD group in the incidence of wound infection (OR = 1.00; 95% CI, 0.46–2.17; *P* = 0.99) (Figure 5).

#### 2.8.4. Incidence of Endocrine Insufficiency

**Figure 6:**
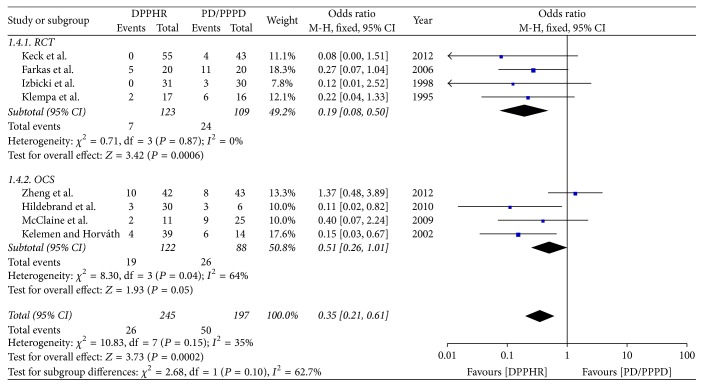
Meta-analysis of the incidence of endocrine insufficiency.

Eight included studies reported the incidence of postoperative** e**ndocrine insufficiency. In these included studies, endocrine pancreatic functions were estimated at least 6 months after operation. We pooled data from the eight studies to compare DPPHR group with PD/PPPD group. The results of meta-analysis show that there is statistical difference between two groups in the rate of postoperative endocrine insufficiency (OR = 0.35; 95% CI, 0.21–0.61; *P* = 0.0002; *I*^2^ = 35% for heterogeneity). Therefore, using a fixed model. The meta-analysis of RCTs and OCS subgroup reveals the incidence of postoperative endocrine insufficiency was statically different between two groups [RCTs (*I*^2^ = 0%, OR = 0.19; 95% CI, 0.08–0.50; *P* = 0.0006), OCS (*I*^2^ = 64%, OR = 0.51; 95% CI, 0.26–1.01; *P* = 0.05)] (Figure 6).

#### 2.8.5. Incidence of Exocrine Insufficiency

**Figure 7:**
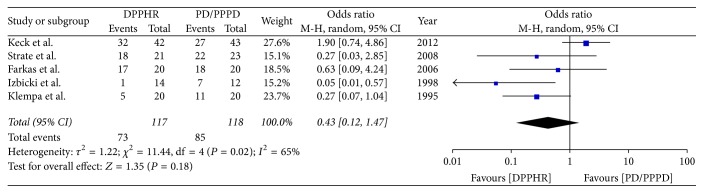
Meta-analysis of the incidence of exocrine insufficiency.

Five included studies reported the rate of exocrine insufficiency after PD/PPPD and DPPHR. Pancreatic exocrine insufficiency was defined as diarrhea and steatorrhea, which improved with pancreatic enzyme replacement. *I*^2^ (*I*^2^ = 65%) revealed obvious heterogeneity among these studies; therefore random model was applied. There was no significant difference between DPPHR and PD/PPPD group in exocrine insufficiency (OR = 0.43; 95% CI, 0.12–1.47; *P* = 0.18) (Figure 7).

#### 2.8.6. Postoperative Weight Gain

**Figure 8:**
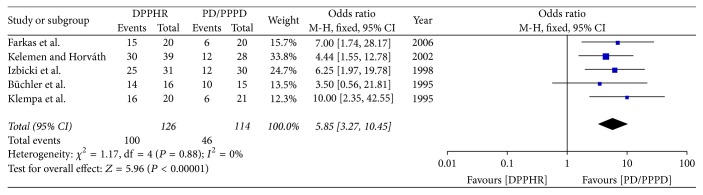
Meta-analysis of the incidence of postoperative weight gain.

Five included studies reported the rate of weight gain after PD/PPPD and DPPHR. In these included studies, the rate of weight gain was estimated at least 6 months after operation. *I*^2^ revealed no obvious heterogeneity among these studies. Therefore, using a fixed model, there was significant difference between DPPHR and PD/PPPD group in postoperative weight gain (OR = 5.85; 95% CI, 3.27–10.45; *P* < 0.00001) (Figure 8).

#### 2.8.7. Incidence of Delayed Gastric Emptying

**Figure 9:**
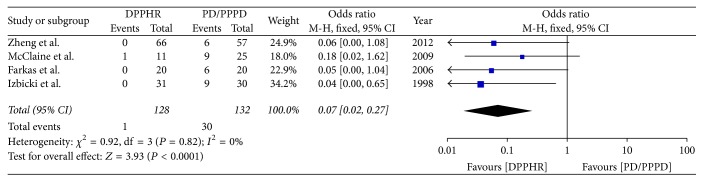
Meta-analysis of the incidence of delayed gastric emptying.

Four studies reported the incidence of delayed gastric emptying after DPPHR and PD/PPPD; there was no significant heterogeneity among studies (*I*^2^ = 0%); therefore fixed model was applied. The result (OR = 0.07; 95% CI, 0.02–0.27; *P* < 0.0001) indicates that there was statistical difference between two groups (Figure 9).

#### 2.8.8. Postoperative Hospitalization Time

**Figure 10:**
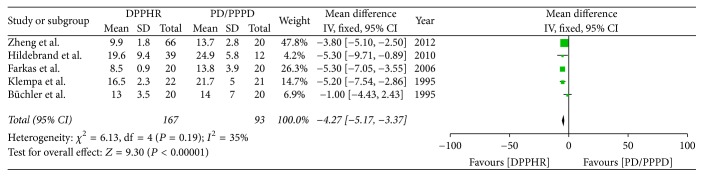
Meta-analysis of the postoperative hospitalization time.

Five included studies reported the postoperative hospitalization time of PD/PPPD and DPPHR. *I*^2^ (*I*^2^ = 35%) revealed no obvious heterogeneity among these studies; therefore, using a fixed model, there was significant difference between DPPHR and PD/PPPD group in hospital time (OR = −4.27; 95% CI, −5.17–3.37; *P* < 0.00001) (Figure 10).

#### 2.8.9. Postoperative Mortality

**Figure 11:**
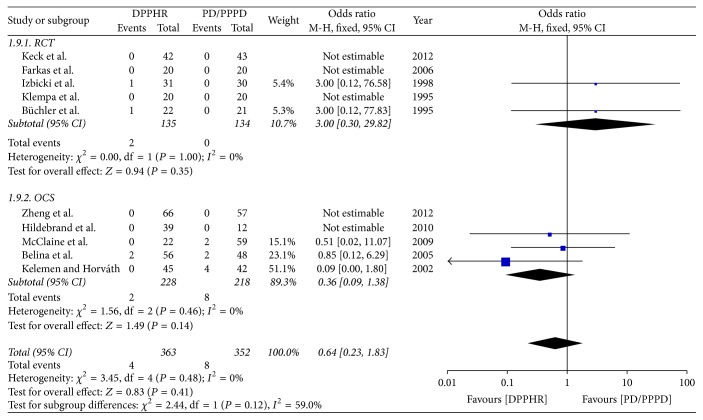
Meta-analysis of the postoperative mortality.

Ten included studies reported the incidence of postoperative mortality, The results of meta-analysis show that there is no difference between DPPHR and PD/PPPD group in the incidence of postoperative mortality (OR = 0.64; 95% CI, 0.23–1.83; *P* = 0.41). The meta-analysis of RCTs and OCS subgroup reveals both no obvious heterogeneity among studies and no obvious difference in the incidence of postoperative morbidity [RCTs (*I*^2^ = 0%, OR = 3.00; 95% CI, 0.30–29.82; *P* = 0.35), OCS (*I*^2^ = 0%, OR = 0.36; 95% CI, 0.09–1.38; *P* = 0.14)], so fixed model was applied (Figure 11).

#### 2.8.10. Postoperative Functioning Scale Scores

**Figure 12:**
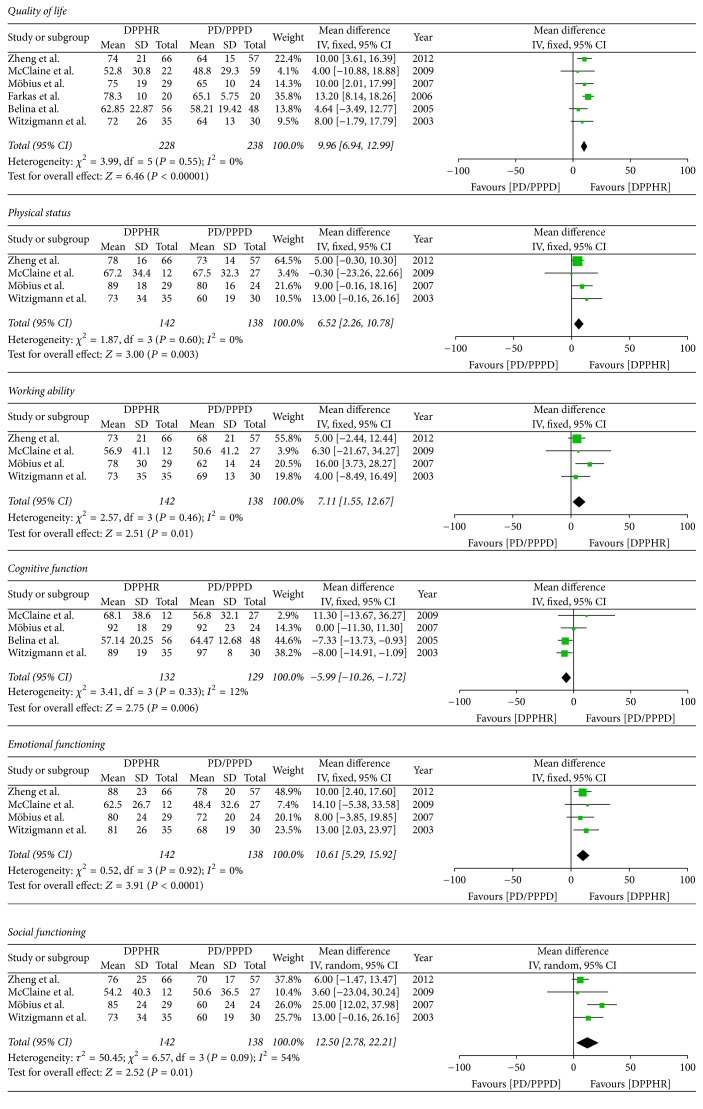
Meta-analysis of the postoperative functioning scale scores.

Another objective criterion in addition to the above basic parameters of a specific surgical method is currently global improvement of the quality of life. The objective outcome assessment of surgical treatment has been made by the EORTC QLQ, used for patients with CP; the system of EORTC QLQ-C-30 includes five terms, such as physical status, working ability, emotional functioning, cognitive functioning, and social functioning. Six included studies reported postoperative functioning scale scores; we pooled data from the six studies comparing two groups. The results of meta-analysis show that there is obvious difference between two groups in quality of life (OR = 9.96; 95% CI, 6.94–12.99; *P* < 0.00001; *I*^2^ = 0% for heterogeneity), physical status (OR = 6.52; 95% CI, 2.26–10.78; *P* = 0.003; *I*^2^ = 0% for heterogeneity), working ability (OR = 7.11; 95% CI, 1.55–12.67; *P* = 0.01; *I*^2^ = 0% for heterogeneity), emotional functioning (OR = 10.61; 95% CI, 5.29–15.92; *P* < 0.001; *I*^2^ = 0% for heterogeneity), cognitive functioning (OR = −5.99; 95% CI, −10.26–1.72; *P* = 0.006; *I*^2^ = 12% for heterogeneity), and social functioning (OR = 10.50; 95% CI, 2.78–22.21; *P* = 0.01; *I*^2^ = 54% for heterogeneity) (Figure 12).

#### 2.8.11. Subgroup Analysis

**Figure 13:**
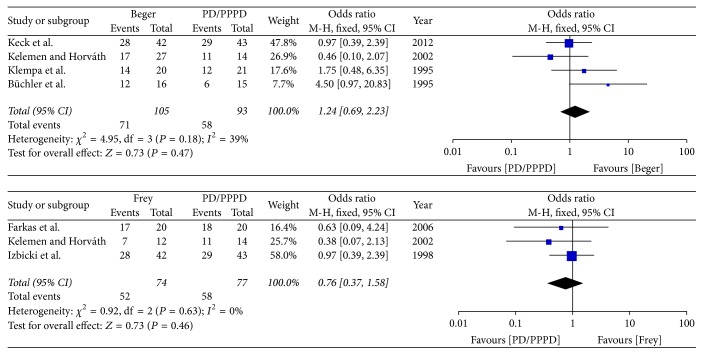
Subgroup analysis of pain relief.

**Figure 14:**
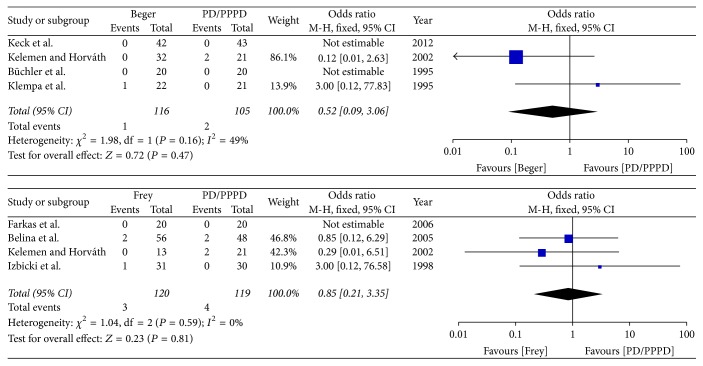
Subgroup analysis of postoperative mortality.

**Figure 15:**
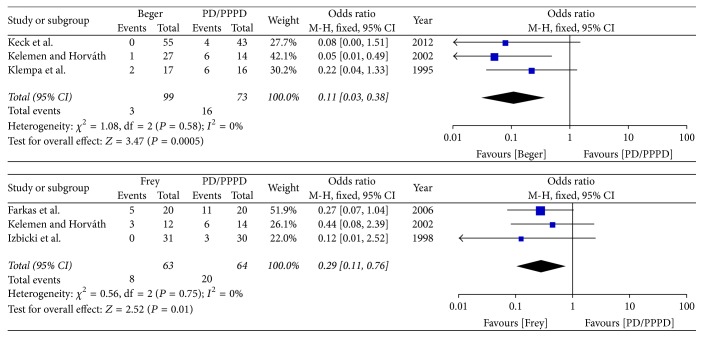
Subgroup analysis of incidence of endocrine insufficiency.

The procedures of duodenum-preserving pancreatic head resection included the Beger and Frey two methods of surgery. Beger procedure was performed with jejunal Roux-en-Y loop which drained the residual pancreas via an end-to-end or end-to-side pancreaticojejunostomy to the body of the pancreas and a side-to-side pancreaticojejunostomy to the excavated pancreatic head remnant and opening intrapancreatic choledochus and suturing the incision to bilateral pancreas tissues. Frey procedure included local resection of the enlarged pancreatic head and a longitudinal incision of the dilated duct; reconstruction was accomplished by longitudinal pancreaticojejunostomy and the common bile duct was drained by cholangiojejunostomy. In order to reduce the heterogeneity resulting from different surgery method, we perform subgroup analysis according to the surgery method Beger or Frey.

(*1) Subgroup Analysis of Pain Relief*. The subgroup analysis result shows that neither Beger nor Frey was significantly different with PD in the rate of pain relief [Beger versus PD (*I*^2^ = 39%, OR = 1.24 95% CI 0.69–2.23; *P* = 0.47), Frey versus PD/PPPD (*I*^2^ = 0%, OR = 0.76, 95% CI 0.37–1.58; *P* = 0.46)]; therefore the fixed effects model was used (Figure 13).

(*2) Subgroup Analysis of Postoperative Mortality*. The subgroup analysis result shows that neither Beger nor Frey was significant different with PD/PPPD in postoperative mortality [Beger versus PD/PPPD (*I*^2^ = 49%, OR = 0.52 95% CI, 0.09–3.06; *P* = 0.47), Frey versus PD/PPPD (*I*^2^ = 0%, OR = 0.85 95% CI, 0.21–3.35; *P* = 0.81)]; therefore the fixed effects model was used (Figure 14).

(*3) Subgroup Analysis of the Incidence of Endocrine Insufficiency*. The subgroup analysis result shows that both Beger and Frey were significantly different with PD/PPPD in postoperative incidence of endocrine insufficiency [Beger versus PD/PPPD (*I*^2^ = 0%, OR = 0.11 95% CI, 0.03–0.38; *P* = 0.0005), Frey versus PD/PPPD (*I*^2^ = 0%, OR = 0.29 95% CI, 0.11–0.76; *P* = 0.01)]; therefore the fixed effects model was used (Figure 15).

#### 2.8.12. Publication Bias

**Figure 16:**
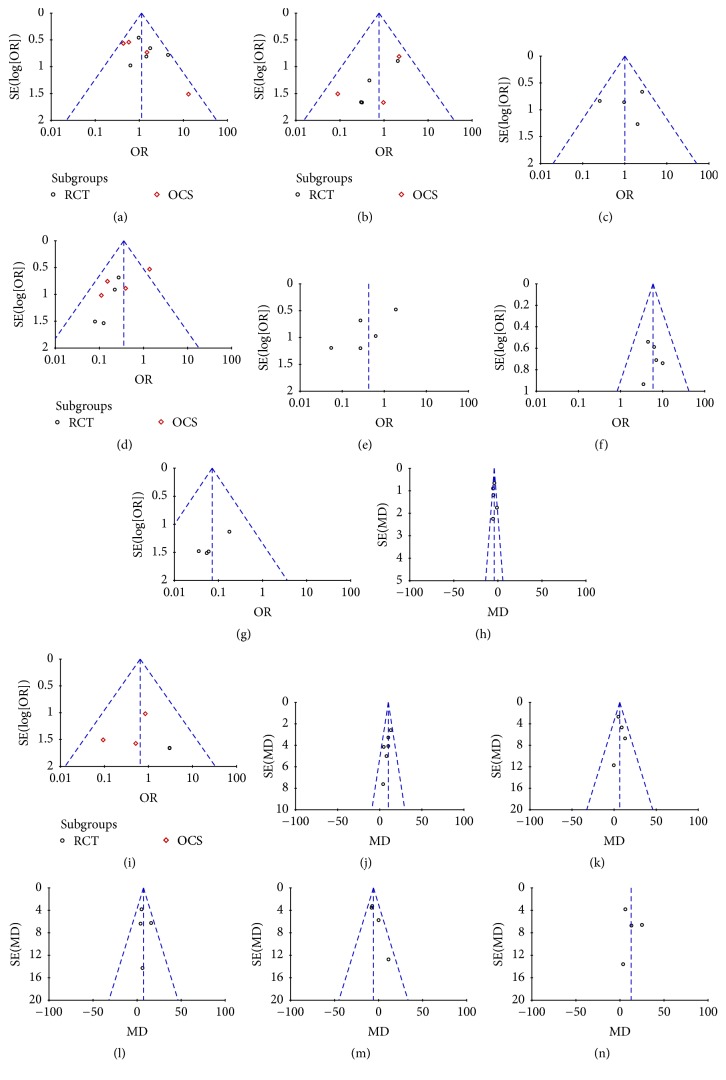
Funnel plots: (a) rate of pain relief, (b) incidence of pancreatic fistula, (c) wound infection, (d) endocrine insufficiency, (e) exocrine insufficiency, (f) weight gain, (g) delayed gastric emptying, (h) postoperative hospitalization time, (i) postoperative mortality, (j) quality of life, (k) physical status, (l) working ability, (m) cognitive function, and (n) social functioning.

**Figure 17:**
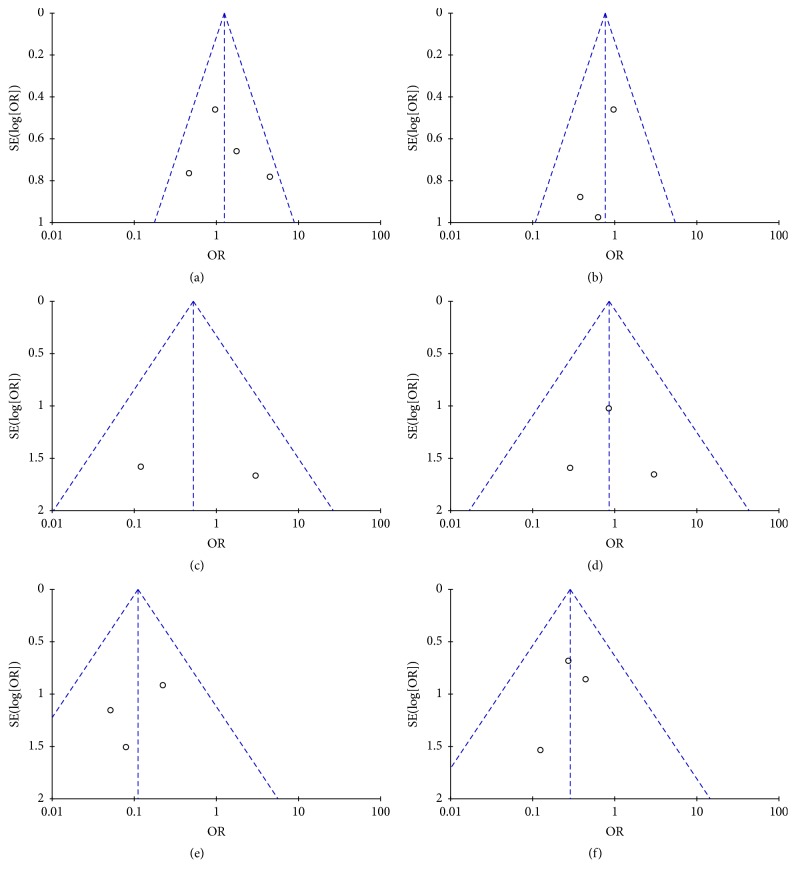
Subgroup funnel plots: (a) rate of Pain relief (Beger), (b) rate of pain relief (Frey), (c) postoperative mortality (Beger), (d) postoperative mortality (Frey), (e) postoperative endocrine insufficiency (Beger), and (f) postoperative endocrine insufficiency (Frey).

Funnel plots were created to assess the publication bias in our meta-analysis of included studies. In the absence of publication bias, it assumes that studies with high precision will be plotted near the average, and studies with low precision will be spread evenly on both sides of the average, creating a roughly funnel-shaped distribution. Deviation from this shape can indicate publication bias. There was no evident asymmetry in the funnel plots (Figures 16 and 17), suggesting a low probability of publication bias.

## 3. Discussion

CP requires conservative treatment [23]. Complications such as bile duct stenosis as well as duodenal, pancreatic duct, or vascular obstruction often necessitate surgical intervention for CP with PH enlargement. Elective surgical procedures undertaken in CP patients can be divided into resection or drainage procedures. The Whipple procedure is first-line therapy for PH tumors. To ascertain if preservation of the duodenum benefits patients, some randomized studies have been done to compare the morbidity, mortality, pain relief, and exocrine/endocrine function between these two surgical approaches.

The result of our meta-analysis showed that PD/PPPD and DPPHR were not significantly different with regard to pain relief, pancreatic fistulae, infection, or postoperative mortality. In CP, pain mechanisms in patients with chronic pancreatitis are incompletely understood and probably multifactorial. Many factors, such as pancreatic duct obstruction, neuropathic changes, alterations in nociception, maybe link to pancreatic pain. Conservative and endoscopic therapy will have less benefit in pain relief, while DPPHR and PD/PPPD are all effective treatments to relieve pain [24]. DPPHR and PD/PPPD were equally effective in controlling pain and had an acceptable low mortality rate. The main purpose of DPPHR is to preserve the integrity of the digestive tracts.

Our results revealed DPPHR to have a low prevalence of delayed gastric emptying compared with PD. We hypothesize that preservation of continuity of the gastroduodenal passage is important for reducing the risk of delayed gastric emptying. DPPHR could also reduce the duration of postoperative hospitalization significantly and reduce the expense of treatment. The longer length of stay in PD was likely attributable to the higher incidence of delayed gastric emptying. Moreover, our group meta-analysis and subgroup meta-analysis revealed that DPPHR could lower the prevalence of endocrine inefficiency compared with PD, whereas there was no significant difference between DPPHR and PD in the prevalence of exocrine inefficiency. Approximately 40–60% of the pancreas is resected in PD compared with 10–40% in DPPHR, so the latter can preserve more normal pancreatic tissue and protect exocrine and pancreatic functions. Compared with PD, DPPHR could increase postoperative weight gain significantly. Indeed, some researchers have reported that 80–90% of patients can increase weight after DPPHR.

Surgical treatment for CP can lower the risk of pain and complications, but also improve the quality of life, physical status, and social and occupational rehabilitation. The European Organization for Research and Treatment of Cancer (EORTC) Quality of Life Questionnaire (QLQ) is a suitable and reliable tool for assessing the global QoL of CP patients [25]. The EORTC QLQ-C-30 comprises five terms: working ability, physical status, cognitive function, emotional function, and social function. Our meta-analysis showed that DPPHR had more advantages than PD in terms of improving QoL. Compared with PD, DPPHR was associated with less damage to the retroperitoneal nerve plexus. Moreover, DPPHR preserves the secretion function of the antrum and duodenum, which benefits postoperative recovery. Our meta-analysis suggests that DPPHR should be adopted as a new standard procedure in the treatment of pancreatic head complications in chronic pancreatitis.

A major limitation of our meta-analysis was that only a small number of high-quality RCTs was included. The surgical experience and methods used at different hospitals and specialist centers could have produced different outcomes and increased the heterogeneity between the included studies. Also, the treatment of complications may have affected the outcome of the RCTs and OCS included in this meta-analysis.

## 4. Conclusions

Our meta-analysis revealed that DPPHR was more beneficial than PD/PPPD in reducing the prevalence of delayed gastric emptying; endocrine insufficiency; duration of postoperative hospitalization. Also, DPPHR increased the QoL of patients. No significant differences were found with regard to the prevalence of pain relief, pancreatic fistulae, wound infection, exocrine insufficiency, or mortality between the two approaches. A similar prevalence of mortality for DPPHR and PD/PPPD was not surprising given their comparable degree of surgical complexity. Therefore, DPPHR should be established as first-line treatment because of lower level of severe early postoperative complications, maintenance of endocrine pancreatic functions, shortening of postoperative hospitalization time, and increase of quality of life compared to pancreaticoduodenectomy.

## Figures and Tables

**Table 1:** The characteristics of all the included studies.

Author	Year	Country	Study type	Group	Patients number	Male/female	Age, y	Study quality
								RCT(jadad system)
								retro(NOS system)
Keck et al. [8]	2012	Germany	RCT	Beger	42	35/7	41.2	5
				PD/PPPD	43	37/6	42.7	
Strate et al. [9]	2008	Germany	RCT	Frey	31	25/6	43.1 ± 6.5	5
				PD/PPPD	30	26/4	44.6 ± 5.3	
Müller et al. [10]	2008	Germany	RCT	Beger	20	18/2	43.0 ± 9.0	5
				PD/PPPD	20	18/2	46.0 ±11.0	
Farkas et al. [11]	2006	Germany	RCT	Frey	20	15/5	43.0 ± 5.0	5
				PD/PPPD	20	15/5	45.0 ± 8.0	
Helmut et al. [12]	2002	Germany	RCT	Beger	35	—	43	7
				PD/PPPD	30	—	48	
Izbicki et al. [13]	1998	Germany	RCT	Frey	31	19/9	43.0 ± 5.0	5
				PD/PPPD	30	19/10	45.0 ± 8.0	
Klempa et al. [14]	1995	Germany	RCT	Beger	22	—	—	5
				PD/PPPD	21	—	—	
Büchler et al. [15]	1995	Germany	RCT	Beger	20	25/9	43.0 ± 9.0	7
				PD/PPPD	20	12/8	46.0 ± 11.0	
Zheng et al. [16]	2012	China	Retro	DPPHR	66	21/16	46.0 (8.8)	7
				PD/PPPD	57	23/14	45.6 (9.7)	
Hildebrand et al. [17]	2010	Germany	Retro	Frey	22	8/14	46.6 ± 9.1	7
				PD/PPPD	59	26/33	54.1 ± 9.7	
McClaine et al. [18]	2009	America	Retro	DPPHR	22	8/14	44.9 ± 11.1	9
				PD/PPPD	59	26/33	46.8 ± 11.1	
Möbius et al. [19]	2007	Germany	Retro	DPPHR	29	—	—	7
				PD/PPPD	24	—	—	
Belina et al. [20]	2005	Czech	Retro	Frey	48	39/9	45 (23–71)	7
				PD/PPPD	56	55/1	48 (29–71)	
Witzigmann et al. [21]	2003	Germany	Retro	DPPHR	38	28/10	42 ± 10	9
				PD/PPPD	32	25/7	47 ± 12	
Kelemen and Horváth [22]	2002	Japan	Retro	Beger	32	13/0	45.3 (36–64)	7
				Frey	13	26/6	45.9 (36–58)	
				PD/PPPD	21	19/2	48.2 (31–70)	

RCT: randomized controlled trial, PD/PPPD: pancreaticoduodenectomy, and DPPHR: duodenum-preserving pancreatic head resection. Jadad scale system: The Jadad scale, sometimes known as Jadad scoring or the Oxford quality scoring system, is a procedure to independently assess the methodological quality of a clinical trial. It is the most widely used assessment in the world. The Newcastle-Ottawa System: the quality of the nonrandomized studies was assessed by using this system. The quality of the studies was evaluated by examining three items: patient selection, comparability of groups, and assessment of outcome.
